# Performance of plasma biomarkers for diagnosis and prediction of dementia in a Brazilian cohort

**DOI:** 10.1002/alz.095754

**Published:** 2025-01-09

**Authors:** Luis E Santos, Paulo Mattos, Thaís Pinheiro, Ananssa Silva, Claudia Drummond, Felipe K. Sudo, Fernanda Barros‐Aragão, Bart Vanderborght, Carlos O Brandão, Sergio T Ferreira, Fernanda Tovar‐Moll, Fernanda G De Felice, IDOR Memory Clinic Initiative

**Affiliations:** ^1^ D'Or Institute for Research and Education, Rio de Janeiro, RJ Brazil; ^2^ Federal University of Rio de Janeiro, Rio de Janeiro, RJ Brazil; ^3^ Neurolife, Rio de Janeiro, RJ Brazil; ^4^ Queen’s University, Kingston, ON Canada

## Abstract

**Background:**

Dementia is a growing concern throughout the developing world and is severely underdiagnosed among the Brazilian population. Despite remarkable progress in the biomarker field in recent years, local testing and validation of plasma biomarkers of AD and dementia is still lacking in Brazil and Latin America.

**Method:**

In this longitudinal cohort study of 145 participants, the diagnostic performance of plasma biomarkers was assessed based on clinical diagnosis and CSF biomarker positivity. Follow‐up data of up to 4.7 years were used to determine biomarker performance in predicting diagnostic conversions. The study was conducted at the Memory Clinic at the D’Or Institute for Research and Education (IDOR) in Rio de Janeiro. Participants were volunteers referred to the service. All were native Brazilians, had Portuguese as their first language and 60+ years of age. They were diagnosed (DSM‐5 criteria) and underwent extensive psychiatric and laboratory assessments. Diagnoses outside the scope of the study were excluded. CSF biomarker data was available for 34% of the sample. Participants were categorized as cognitively normal controls (n = 49), amnestic mild cognitive impairment (aMCI; n = 29), Alzheimer’s disease (AD; n = 37), Lewy body dementia (n = 23), or vascular dementia (n = 7). Plasma samples collected at initial and follow‐up visits were tested for relevant biomarkers.

**Result:**

Plasma Tau, Aβ_40_, Aβ_42_, NfL, GFAP, pTau231 and pTau181 were measured on the SIMOA HD‐X platform. Results were evaluated against clinical diagnosis and CSF biomarker status. Plasma NfL and GFAP could discriminate between all‐cause dementia and controls with ROC AUCs of 0.79 (95% CI: [0.70–0.87]) and 0.74 [0.65–0.83], respectively. Plasma pTau181 had good diagnostic performance discriminating clinical AD (AUC = 0.89 [0.82–0.96]), CSF‐biomarker+ aMCI/AD (AUC = 0.90 [0.82–0.98]), or CSF‐biomarker‐confirmed AD (AUC = 0.95 [0.89–1.00]) from controls.

**Conclusion:**

In this first description of the plasma biomarker profile of a Brazilian dementia cohort, plasma pTau181 confirmed its potential as a clinically useful diagnostic tool. This study comprises an initial step towards local validation and adoption of dementia biomarkers in Brazil and Latin America.

